# Meteorological Table

**Published:** 1813-11

**Authors:** 


					433
METEOROLOGICAL TABLE.
From September 26, to October 25, 1813.
D.
?
o
ki
Therm.
56 62
55 63
56 60
55 59
55 50
50 57
52 56
53 58
55 57
61 63
60 64
68 63
55 59
58 60
55 59
52 55
48 56
57 54
43 46
13 49
44 47
48 53
43 50
39 47
42 48
50 54
52 56
55 57
39 48
41 54
Barom.
301 ?
301 30
30 1
302 ?
302 ]
29? f
29' '
298 ?
29* ''
29s -
29? *
29
29
29
29
29
29
29
29
29
293 28?
28* 292
294 6
297 ?
297 ?
296 7
30 ?
299 30
Hygrom.
Dry. Damp.
13 12
15 12
? 15
26 19
? 15
? 2 ?
Winds. I Atmos. Variation.
? 10
25 15
16 12
20 35
27 32
30 ?
15 2
13 ?
15 ?
27 ?
21 9
19 ?
20 10
19 ?
15 ?
16 9
16 10
21 30
12 17
20 18
20 lo
12 6
IE..
NK.j
NR.
NE.
f!..
IE..
E,.
IE.SE.
25 SE.
sw. ,w?
svv.s.
s.
sw...
NW..
w..
\v....
vv..
NW..
NW.
SW...
20jSW..SE.
15 W. .NE..
11|NW..
16 NE..
20 NE..
17 E..
24]SE..
20 SE..
15jSE..
10! E.
C... F... C..
F... ?.... C..
Fog.. F.. C...?.
Fog .F..C...-R. in Nj
C... R. C...
F....C..?..a...inN|
f.7. c.7f..
C..?...?...R....inNf
R... ?....? .-. F.
F...R.. ?.. C....|
C.... F"....
R...F... C..R...
R..F..
F...C...R...?....inNi
C...F..?...R... inN I
F.. ? ... ? ....
F.. R.. ?... F..
F. R... ?... C...
F.. R... ?.. F..
F C...
F... ?i... C..
Fog ...C..
R... ? ..
Fog... C...
Fog... C..F.
Fog.. C..
F.. R. Fog ..
i
Quantity of rail) from the 26th of September to the 25th of October, three
inches ?j-6q?5. On the 6th, storm of thunder, with heavy rain in the night.
16ih, serene lightning, very frequent in the evening. This interval has been
unusually wet for the season of year. The equinoctial gales, which had
not appeared at the accustomed time, have blown with considerable violence
in October.
Inflammatory affections of the chest, especially in children, have been
frequent; and Scarlatina has spread rapidly in some districts.
October 26th.

				

